# PVD growth of spiral pyramid-shaped WS_2_ on SiO_2_/Si driven by screw dislocations

**DOI:** 10.3389/fchem.2023.1132567

**Published:** 2023-03-03

**Authors:** Yassine Madoune, DingBang Yang, Yameen Ahmed, Mansour M. Al-Makeen, Han Huang

**Affiliations:** ^1^ Hunan Key Laboratory of Super-microstructure and Ultrafast Process, School of Physics and Electronics, Central South University, Changsha, China; ^2^ Department of Electrical and Computer Engineering, University of Victoria, Victoria, BC, Canada

**Keywords:** spiral patterns, tungsten disulfide, physical vapor deposition, Raman spectra, screw-dislocation driven growth

## Abstract

Atomically thin layered transition metal dichalcogenides (TMDs), such as MoS_2_ and WS_2_, have been getting much attention recently due to their interesting electronic and optoelectronic properties. Especially, spiral TMDs provide a variety of candidates for examining the light-matter interaction resulting from the broken inversion symmetry, as well as the potential new utilization in functional optoelectronic, electromagnetic and nanoelectronics devices. To realize their potential device applications, it is desirable to achieve controlled growth of these layered nanomaterials with a tunable stacking. Here, we demonstrate the Physical Vapor Deposition (PVD) growth of spiral pyramid-shaped WS_2_ with ∼200 μm in size and the interesting optical properties *via* AFM and Raman spectroscopy. By controlling the precursors concentration and changing the initial nucleation rates in PVD growth, WS_2_ in different nanoarchitectures can be obtained. We discuss the growth mechanism for these spiral-patterned WS_2_ nanostructures based on the screw dislocations. This study provides a simple, scalable approach of screw dislocation-driven (SDD) growth of distinct TMD nanostructures with varying morphologies, and stacking.

## Introduction

Two-dimensional (2D) layered transition metal dichalcogenides (TMDs) have emerged as promising candidates for optoelectronic devices due to a plentiful choice of materials (Mak et al., 2010) (Xiao et al., 2022) (Yang et al., 2017). Multilayer TMDs display unique optoelectronic properties dependent on the layer number, such as a transition from the indirect band gap in multilayer to the direct band gap in monolayer (Manzeli et al., 2017). The different stacking modes also have critical effects on the properties of TMDs (Fan et al., 2017; Sarma et al., 2019) (Ci et al., 2022). It appeared that polarization enhancement with two petals along staggered stacking direction in 3R MoS_2_ (Shi et al., 2017). The 3R-like TMDs few layers and spiral structures also show high degree of SHG polarization at room temperature due to the inversion symmetry breaking (Ma et al., 2021). Furthermore, bilayer or multilayer TMDs materials with different twisted angles always exhibit a different interlayer coupling to tune the properties of TMDs (Wang et al., 2019).

Spiral TMDs structures display more complex stacking than 2H stacking multilayer structures such as continuously supertwisted stacking, richer physical, and chemical effects (Ci et al., 2022) (Tong et al., 2022). Screw dislocation is a typical line defect for the materials and the growth driven by spiral dislocation is a classic crystal growth mode at relatively low supersaturation (Cain et al., 2016) (Forticaux et al., 2015) (Meng et al., 2013). Many methods have been developed for the fabrication of spiral TMDs, including chemical vapor deposition (CVD) and physical vapor deposition (PVD) (Barman et al., 2019) (Wang et al., 2019) (Hao et al., 2016) (Fan et al., 2018). The hybrid spiral-like MoS_2_ crystals with distinctive electrostatic properties have been synthesized through increasing the growth temperature to 1,000°C by CVD method (Hao et al., 2016). The spiral WS_2_ with non-linear optical effects, a high value of valley polarization could be prepared by CVD using a mixture of WO_3,_ and S as precursors (Barman et al., 2019) (Fan et al., 2017). Compared with CVD method, PVD method is simpler, pollution-free as well as can synthesize spiral TMDs which have higher hardness, better thermal stability, and more stable chemical properties. The controllable growth of layer by layer and spiral WS_2_ with ∼30 μm in size has been realized by PVD. It reveals that the number of screw dislocations, orientation of new layer determine the morphologies, and stacking behaviors of the complex spiral nanostructures combined with SHG (Fan et al., 2018). However, it is difficult to synthesize high quality spiral WS_2_ in specific areas, the size of spiral WS_2_ is too small obtained by these methods (∼70 μm ), limiting their applications on non-linear optical effects or electrocatalytic hydrogen evolution, etc. It is still a significant challenge to controllable fabrication for large area, and size of spiral TMDs.

Here, we report a controllable growth of large size (∼200 μm ) spiral patterned WS_2_ with different stackings and elucidate their formation mechanisms. By tuning PVD temperature and precursor supersaturation, we demonstrated the controllable growth of layer by layer (LBL) structure and a series of spiral structures including single, double, and multi-spiral patterns. As the temperature decreasing, the complexity of spiral structures gradually increases. By related atomic force microscopy (AFM) with Raman spectroscopy measurements, we reveal how multiple dislocations, how the orientation of screw dislocations can affect the stacking behaviors, and formation mechanisms of spiral WS_2_.

## Materials and methods

**FIGURE 1:**
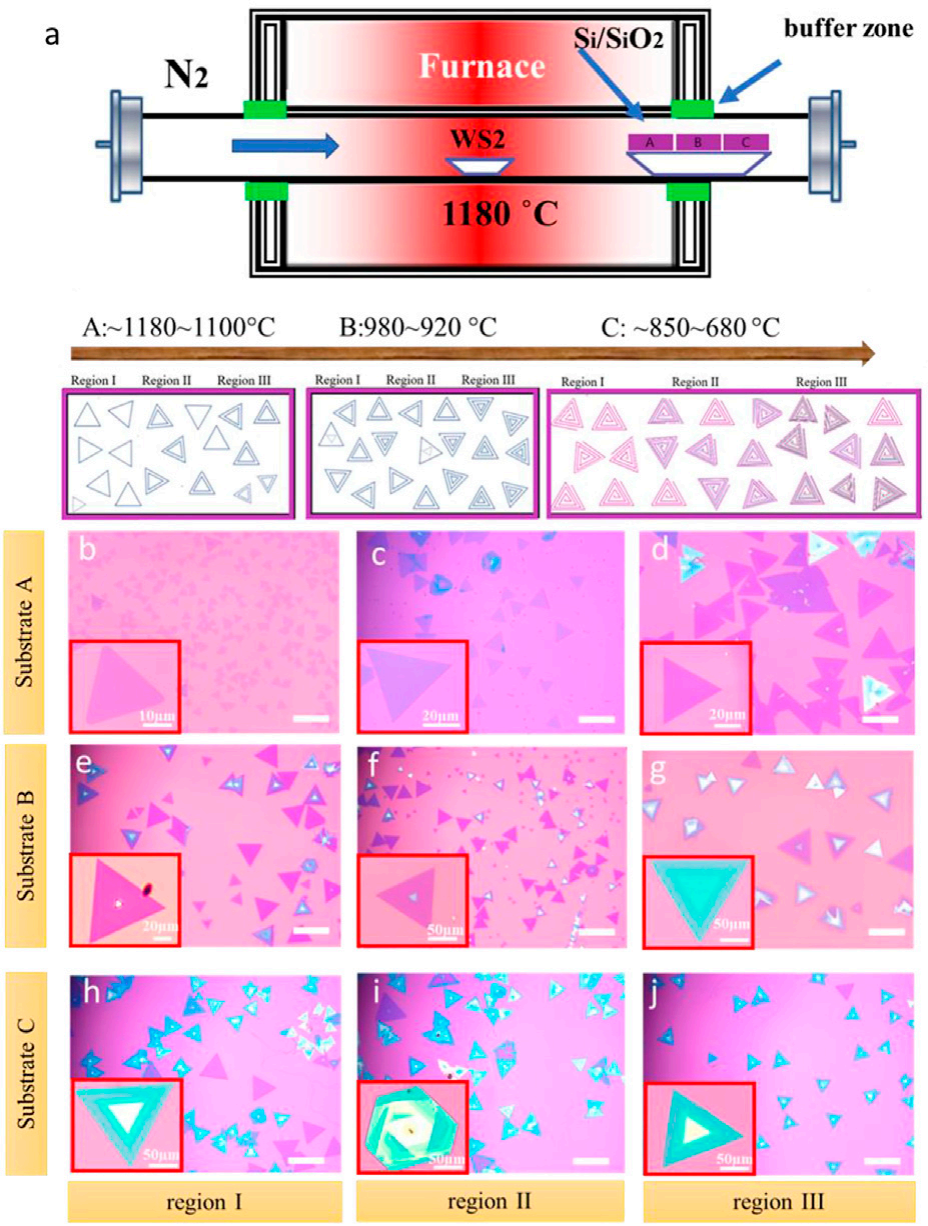
Controllable growth and Optical image of the Layer-By-Layer Structures and Spiral Structures of WS_2_ **(A)** Schematic Illustration of the PVD experiment setup for TMD synthesis. The distribution of WS_2_ from the substrate of **(A–C)**: Monolayers, Bilayers. A few layers, Layer-By-Layer (LBL) and spiral structures. **(B–D)** The optical images of Ⅰ (monolayer), Ⅱ (a mixture of bilayers and trilayer) and Ⅲ (few layers) regions on substrate **(A) (E–G)** The optical images of Ⅰ and Ⅱ (multilayers) and Ⅲ (layer by layer) regions on substrate **(B) (H–J)** The optical images of Ⅰ (single-spiral pattern), Ⅱ (double-spiral patterns) and Ⅲ (multi-spiral patterns) regions on substrate **(C)**.

To synthesize WS_2_ nanoflakes, a ceramic boat filled with WS_2_ (ALDRICH, 99%) powder (0.4 g) was placed at the heating center of a 2-inch quartz tube (2-inch diameter, 60 cm length), a three-piece of SiO_2_/Si (300 nm, 1.5 × 1.5 cm) was placed at 6 cm(A),8 cm (B), and 10 cm (C) downstream from the center of the tube furnace. N_2_ flow was passed into the reaction chamber at a rate of 300 sccm for 10 min to ensure a clean environment for sample synthesis (anhydrous and oxygen-free environment). N_2_ flow of 60 sccm was applied from the source to the substrate during the heating process (target temperature 1,180°C in 70 min) and held for 12 min, then cooled down the PVD tube furnace. To ensure the temperature difference, the three substrates were placed in different areas, substrate A inside the tube with a temperature of 1,180°C, substrate C at the end of the estuary, which is outside of the oven chamber, with a temperature of 850°C–680°C, and the substrate B in the middle of the estuary with a temperature of 920°C–980°C. A gradient in the temperature difference is illustrated in Figure 1A the red color gradually changed from the middle to the edge from dark to light as an indication of the temperature change.

## Results and discussion

We design a PVD method to prepare monolayer (ML), few layers (FL), layer by layer (LBL), and spiral WS_2_, as shown in Figure 1A. The distances between substrates A, B, and C and WS_2_ powder (heating center) are 6 cm, 8 cm, and 10 cm, respectively, where the growth temperature can be controlled well. The growth temperature of substrate A was 1,180°C which was inside the tube, that of substrate C was ∼680°C–850°C which was at the end of the estuary, nearly outside the oven chamber, and that of substrate B was ∼920°C–980°C which was in the middle of the estuary. Figures 1B–D show that many monolayers and a few layers WS_2_ were synthesized on substrate A with a high temperature ranging from 1,100°C to 1,180°C. The region Ⅰ of substrate A displays the monolayer WS_2_ with regular triangle and an average size of 30 μm–40 μm as shown in lower-left corner of Figure 1B. The region Ⅱ of substrate A contains a mixture of bi/trilayer WS_2_ with an average size of about 50 μm–60 μm (Figure 1C). The region Ⅲ of substrate A, as shown in Figure 1D, contains multilayers with an average size of about 70 μm–90 μm where the temperature in this area reaches approximately 1,100°C, lower than the Ⅰ region of substrate A. Multilayers and LBL structures are synthesized on substrate B with a temperature ranging from 920°C to 980°C, as shown in Figures 1E–G. For substrate B, the number of layers increases from multilayers in region I to AA or AB stacking in region Ⅱ with an increase in size from 90 μm to 100 μm to 120 μm–150 μm. The LBL structures are synthesized in the region Ⅲ on substrate B with an estimated size of 180 μm–220 μm, which suggests a flat triangular top on a larger and thinner flake. As the temperature decreasing further, the spiral patterns of WS_2_ were observed on substrate C. The structures in region I (Figure 1H) present a single spiral pattern with an average size of ∼220 μm where the temperature is approximately 850°C. Compared to region I, region Ⅱ (Figure 1I) has double spiral patterns with temperatures between 850°C and 680°C. The region Ⅲ (Figure 1J) exhibits more intricate multi-spiral patterns with a temperature near 680°C. The average size of double spiral and multi-spiral patterns is ∼200 μm. Compared with LBL growth above, the size of WS_2_ obtained by spiral growth did not have an obvious difference with the decreasing temperature.

We found that a monolayer is formed at a high temperature with small sizes, that the sizes, the number of layers increase little by little as the temperature decreases to form a LBL, and SDD structures in the end (Morin et al., 2011). With the temperature decreasing, the LBL and the size of spiral WS_2_ reaches ∼220 µm with a total area over 0.3 cm^2^ × 0.3 cm^2^, larger than the largest obtained by CVD techniques at low temperature (∼70 μm) (Yin et al., 2015) (Zhao and Jin, 2020).

The PVD-deposited 2H-WS_2_ nanoplates have a variety of morphologies, including triangles, truncated triangles, hexagons, etc (Figures 1B–J). The surface free energy of the relevant crystal face affects the development rate of the crystal face in 2D crystals, where the surface free energy corresponds to the crystal’s edge free energy. When the free energy is high, the crystal edge is exceedingly unstable. At this point, the free electrons in the free state will be swiftly absorbed to form the edge in a stable state of low free energy, causing the edge of high free energy to develop fast and the edge of low free energy to grow slowly (Park et al., 2017; Wang et al., 2018). As a consequence of the fast growth behavior, the crystal face with high free energy will ultimately get smaller or vanish. On the contrary, the slow-growing crystal face will eventually become the biggest (Li et al., 2018). As a result, the final crystal form will be connected to the development rate of various kinds of edge terminals, which is further influenced by the precursor volume ratio, which directly influences the growth rate of distinct crystal planes. We employed a constant flow in a quartz tube to retain the precursor to establish a stable air flow environment, which guarantees that the WS_2_ vapor is thoroughly volatilized and evenly dispersed throughout the reaction process. As a result, the concentration distribution of WS_2_ precursors (volatilization and diffusion) may be regarded as the primary factor influencing the various form morphologies (Yan et al., 2023).

**FIGURE 2:**
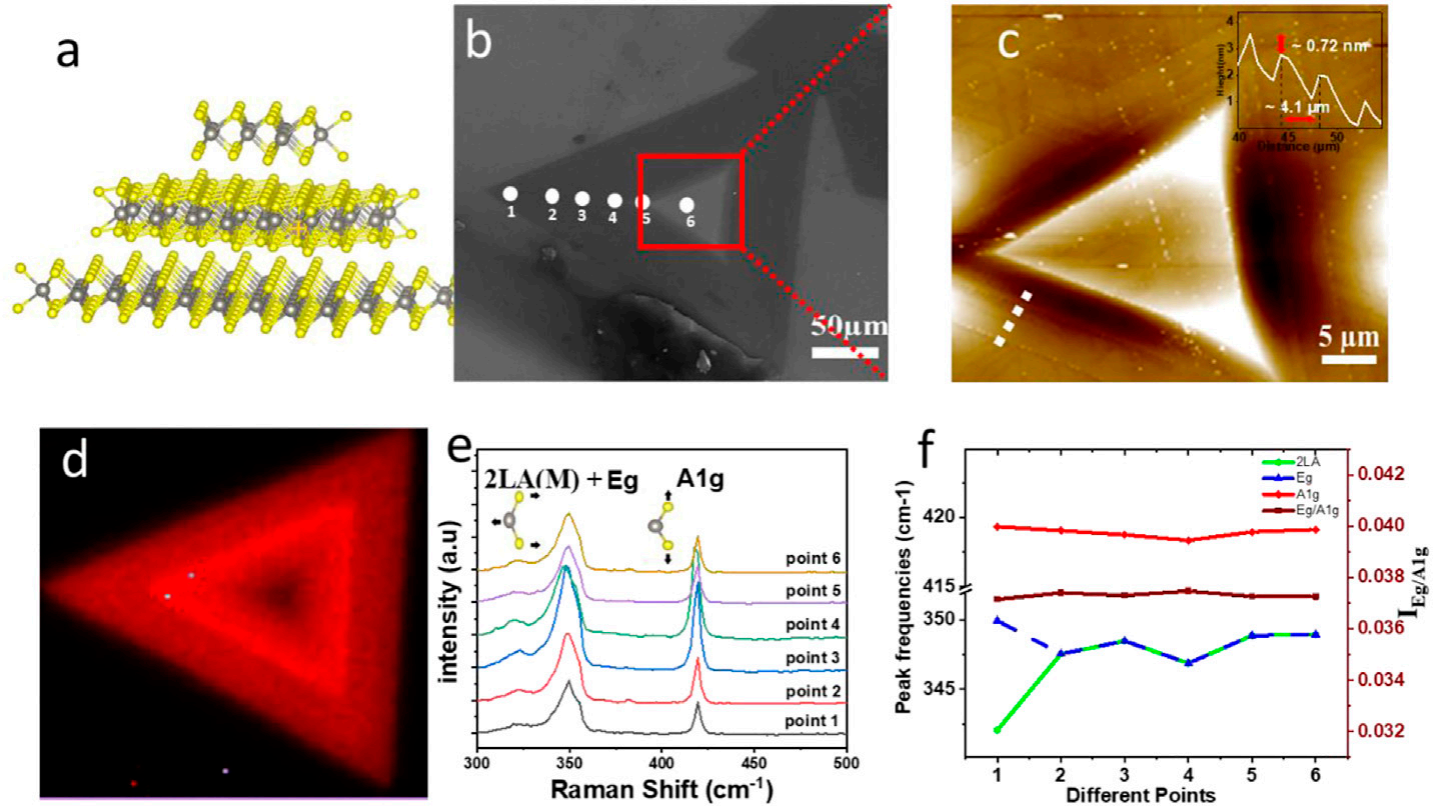
Layer by Layer (LBL) growth. **(A)** Illustrations of the crystal LBL structure. **(B)** SEM image of WS_2_ LBL structure. **(C)** AFM image corresponding to the marked red square in SEM image. **(D)** Raman intensity mapping of $E_{2g}^{1}$ mode (348 cm^-1^). **(E,F)** Raman spectra and peak positions ( $A_{1g}{,E}_{2g}^{1},2LA)$ as well as intensity ratios of $I_{E_{2g}^{1}}$ / $I_{A_{1g}}$ of six points from edge to center, as marked in SEM image.

Figure 2A shows the illustrations of LBL WS_2_ structures. There are many possible WS_2_ stacking configurations but two of the most important are the AB-stacking and the AA-stacking configurations, as shown in Supplementary Figure S2B (Rudenko et al., 2021). Figure 2B shows the SEM images of high-coverage LBL structures in substrate B of region III with a size of 200 μm. Figure 2C shows that the AFM image corresponds to the marked red square in Figure 2B, which confirms the LBL growth of WS_2_. The line profile in Figure 2C indicates that the height increases gradually from edge to center and the height of each step is ∼0.72 nm, consistent with monolayer WS_2_. Figure 2D shows the Raman intensity mapping of $E_{2g}^{1}$ mode (348 cm^-1^). It can be observed that the Raman intensity of LBL structure from the edge to the center first increases then starts to decrease, and the corresponding number of layers gradually increases. The WS_2_ Raman spectra related to the number layers in Figure 2E confirm the above Raman intensity mapping results (Zeng et al., 2013; Qian et al., 2018). The number layers of materials and the intensity of incident light in the scattering process both affect the Raman intensity as mentioned in previous reports (Shao et al., 2020) (Diep et al., 2019).The local electrical field will be significantly less than the incident electrical field for thick WS_2_ materials, which is known as the local field effect for high reflective index TMDCs. Therefore, a faint Raman signal will be seen in thick WS_2_ materials. Furthermore, the local field impact is minimal for extremely thin WS_2_ layers. As a result, there may be a maximum Raman intensity at a certain height in WS_2_. According to a recent study, the first few layers of WS_2_ (bilayers to five layers) have the largest Raman intensity which gradually decrease after the bulk starts to form (Li et al., 2013).

Figure 2F shows that both $A_{1g}$ and $E_{2g}^{1}$ peaks position is not shifted despite the thickness increases (the height of difference between the first and the sixth points is ∼24 nm). This solidification in the two modes is in good agreement with the previous results for MoS_2_ (Yan et al., 2015), and the higher restoring strength brought on by the vdW interactions produced between the layers. We observe that the error in calculating the frequency shift of both the 2LA(M) and $E_{2g}^{1}$ modes rise due to their close proximity. A clear frequency relationship with the number of layers cannot be established since the change in frequency is of the order of the error bar, which is also impacted by the fitting procedure. A shift in dielectric screening with the number of layers is also anticipated for WS_2_ (Jin et al., 2010). Stronger dielectric screening of the long-range coulomb interactions between the effective charges in thicker samples may be the reason for the $E_{2g}^{1}$ mode’s anomalous behavior. We also looked at how the number of layers affected the relative intensity of the strongest Raman signals. With the number of layers increasing, there are no discernible variations in the intensity ratio $I_{E_{2g}^{1}}$ / $I_{A_{1g}}$ (Berkdemir et al., 2013).

**FIGURE 3:**
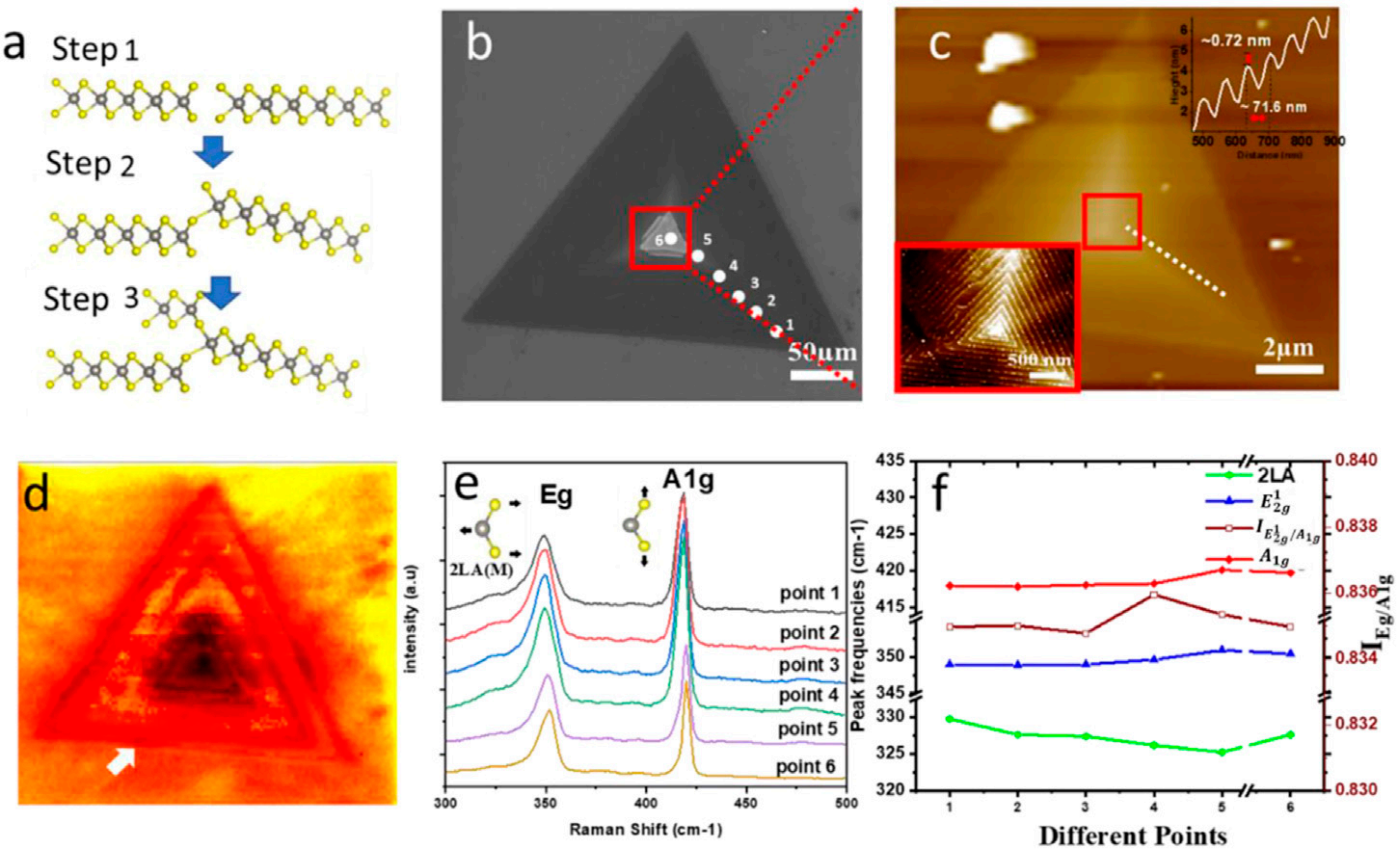
Screw dislocation growth of single spiral. **(A)** Illustrations of the single spiral structure **(B)** SEM image of single spiral structure of WS_2_ **(C)** AFM images corresponding the marked areas in SEM image. **(D)** Raman intensity mapping of $E_{2g}^{1}$ mode (350 cm^-1^). **(E,F)** Intensity ratios and peak frequencies of WS_2_ Raman modes. **(E)** Frequencies of the $\left.{\left(2LA,E\right.}_{2g}^{1},A_{1g}\right)$ Raman modes as a function of thickness (different positions mean different thicknesses) for *λ*_*exe*_ = 633. The error bars correspond to the standard deviations and Each point represents an average over six different positions **(E)** Thickness-dependent intensity ratios of $I_{E_{2g}^{1}}$ / $I_{A_{1g}}$ **(E,F)** of six points from edge to center, as marked in SEM image.

The anisotropic pyramidal growth is continued in this fashion (formed) by self-perpetuating steps of SDD layers. Under mild supersaturation circumstances, screw dislocations form step edges (slipped planes) in the bottom layer (Daher et al., 2018). Because of the large concentration of precursors, the unintentional rising of a grain boundary caused by varying growth rates of multiple edge terminations begins spiral growth, which is schematically shown as step 1 in Figure 3A. The spiral development is also catalyzed by the uneven surface of the substrate, which is created by the partial etching of S_i_O_2_/S_i_ using piranha and plasma treatment. The unsaturated sulfur edges in the slipping plane operate as nucleation sites for additional precursor atom addition, resulting in the formation of the second layer on top of the bottom layer, as illustrated in Figure 3A (steps 2, 3). The growth process continues up to an interface or a nodal point, generating a spiral after the slip plane is produced with vertically mismatched edges (Sarma et al., 2019).

Figure 3B shows a typical SEM image of a single spiral WS_2_ with ∼220 μm, which indicates the atomically flat surface of WS_2_. The zoom-in AFM image of the red square region of the SEM image is shown in Figure 3C, revealing that each step height of a single spiral of WS_2_ is ∼0.72 nm, consistent with the height of monolayer WS_2_, with an estimated height of ∼25 nm (from the edge to the center). When the single spiral WS_2_ is projected onto the 2D basal plane, the layer size progressively decreases from the center to the edge, as shown in Supplementary Figure S4. The low-left corner of Figure 3C; Supplementary Figure S5A as well as Supplementary Figure S5B display AFM images of a single spiral, double spiral, and multi-spiral with clear screw dislocation, respectively. The white lines in Supplementary Figure S5 (near the dislocation center) of the core serve as an example of the included angle, defined as the angle between the traces of the many dislocation spirals that share a common core. There may be two sets of included angles between screw dislocation spirals based on the three-fold symmetry of the monolayer TMD structure. The center structure of spiral nanoplates stays as triangular spiral traces whether the included angles are just (0°C, 360°C) or (120°C, 240°C), as shown in low-left corner of Figure 3C; Supplementary Figure S5. In contrast, the center of those spiral nanoplates shows hexagonal spiral traces when we inspect the second set of included angles of (60°C, 180°C, and 300°C) (Shearer et al., 2017). It should be noted that multi-spiral pattern nanostructures are far less often seen compared to single or double-spiral pattern structures.

Figure 3D shows the Raman intensity (350 cm^−1^) mapping of the spiral structure, revealing a regular variety of $E_{2g}^{1}$ intensities in the region of single spiral WS_2_ (Chen et al., 2017). Also, it can reveal that they are multilayer flakes, which corresponds with AFM measurement shown above. Figure 3E summarizes the single spiral WS_2_ Raman spectra as a function of the thickness, the Raman spectra was measured six consecutive times at each of the 6 points indicated in Figure 3B to calculate the error bars of the Raman shift: $\left[{2LA\left(\pm 0.8\;{cm}^{-1}\right),E}_{2g}^{1}(\pm 0.4\;{cm}^{-1}),A_{1g}(\pm 0.3\;{cm}^{-1}\right.$ )]. The thicknesses, distances between the first and last point are ∼25 nm and 150 μm, respectively, as shown in Supplementary Figure S4.

To determine the frequency dependence of the main WS_2_ Raman peaks ( $E_{2g}^{1}$ and $A_{1g}$ ), we fitted several Lorentzian peaks to each spectrum, as shown in Figure 3F. When the thickness increases, the $E_{2g}^{1},A_{1g}$ modes exhibit minor redshift (Ci et al., 2022). The growing restoring force brought on by vdW contacts that have been developed between layers is compatible with the hardness of the $A_{1g}$ mode. However, the 2LM phonon mode also exhibits subtle blueshifts when the thickness increases (Ci et al., 2022). In screw dislocation (single, double, and multi-spiral patterns) WS_2_ the proximity of 2LA (M) and $E_{2g}^{1}$ increases the error in determining the frequency offset for both modes. Especially since the number of layers is very large, it may reach more than 30 layers at least (in this sample, the thickness is ∼25 nm, every layer ∼0.72 nm). Due to the closeness of the layers to one another, it is a challenge to establish a clear frequency dependence with number of layers. (∼71.6 nm shown in the inset of Figure 3C). A change in buffer shifting is also expected with the number of layers in WS_2_. It may be caused by enhanced and a stronger dielectric sifting of long-range or by coulomb interactions between effective charges in bulk samples (Yin et al., 2014). We also studied the relative intensities of the strongest Raman peaks as a function of thicknesses (different points with different numbers of layers). The most intense features in the Raman spectrum correspond to the $E_{2g}^{1}$ and $A_{1g}$ modes and the intensity ratio $I_{E_{2g}^{1}}$ / $I_{A_{1g}}$ does not show major changes with the number of layers or thickness. It has been generalized by observing different samples of single spiral patterns (Supplementary Figure S6) (Berkdemir et al., 2013).

**FIGURE 4:**
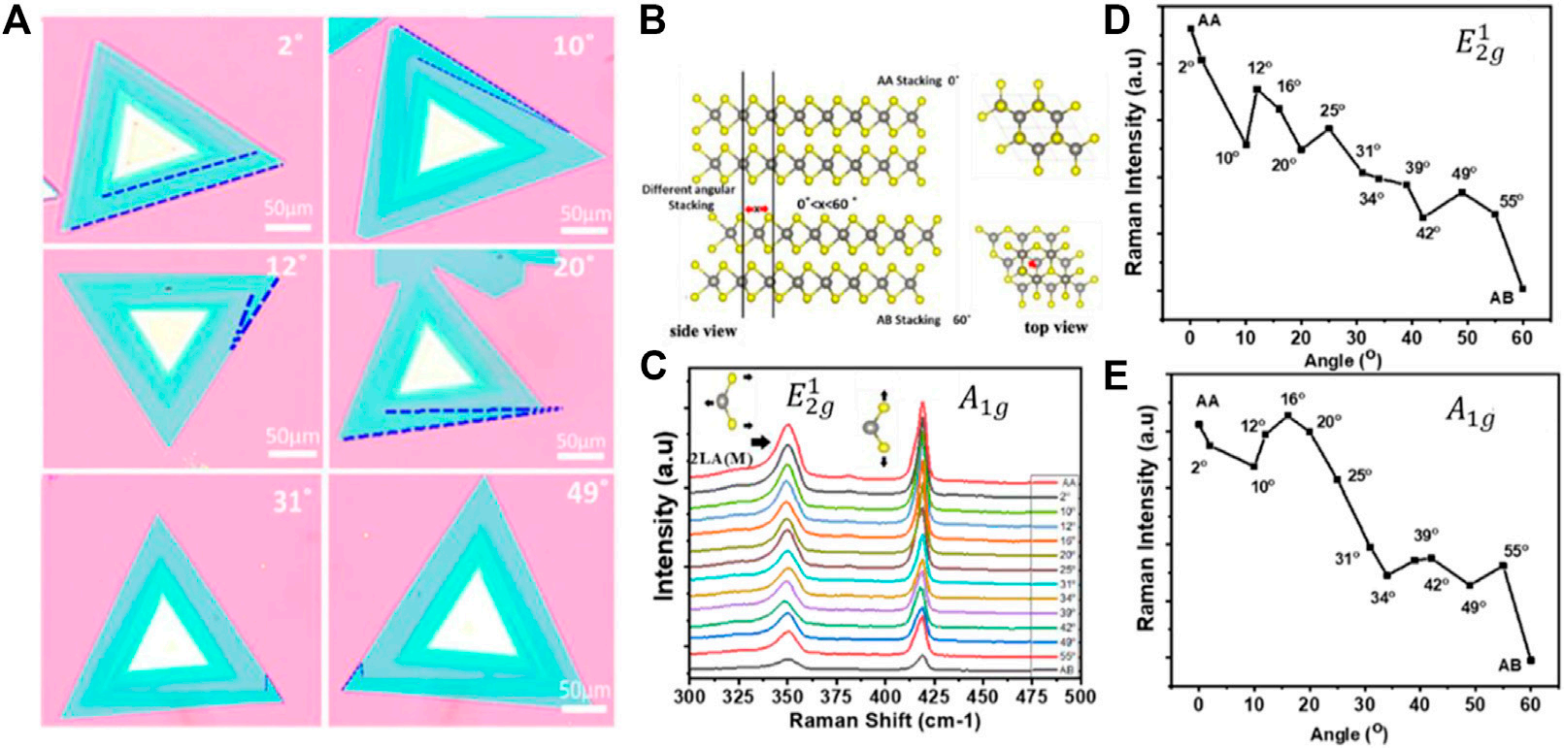
Twist angle-dependent responses bilayer WS_2_ crystals with different twist angles. **(A)** Optical images and **(B)** atomic structure of different angles hetero-bilayer stacking WS_2_ in a 2 × 2 × 1 supercell from a side view (upper panel) and top view (lower panel), respectively. **(C)** Raman intensity at different twist angles on single spiral bilayer WS_2_. **(D,E)** Twist angular dependence of Raman intensity ( $E_{2g}^{1}$ mode $A_{1g}$ mode).

Figure 4A shows the Optical images of WS_2_ bilayers with varying twist angles of 2°C,10°C, 12°C, 20°C, 31°C, and 49°C. As a result of the steric repulsion effect, the interlayer coupling in the randomly twisted bilayer was lower than in LBL stacking (AA stacking) (Zhang et al., 2020). Spin-orbit coupling would find a wider range of uses in angle-dependent moiré excitons, spintronics, and valley electronics if the twist angles were varied. It is depicted by the atomic structure schematic of LBL, single spiral patterns hetero-bilayer WS_2_ in a 2 × 2 × 1 supercell from a top perspective (Ghatak et al., 2020) (lower panel), and side view (upper panel) in Figure 4B, respectively. The variable interlayer coupling may be further confirmed by the twist angle-dependent optical responses by accumulating Raman spectra at different angles (Figure 4C). The characteristic peak intensities ( $E_{2g}^{1}$ and $A_{1g}$ ) of WS_2_ in the Raman spectra steadily decrease periodically as the twist angle increasing, showing that the mechanical coupling effect was typically less due to the stacking angle in Figure 4D, E (Shao et al., 2020).

Interlayer coupling interactions of SDD (single spiral) differ in randomly twisted bilayers compared to LBL due to the upper and lower layers of multi-spiral pattern nanostructures being far less often seen compared to single or double-spiral pattern structure saving different arrangements of atoms. The majority of 2H-WS_2_ nanosheets with terraced or spiral structures are dispersed across the substrate, according to the morphological information of 2H-WS_2_ nanosheets. So, the moiré superlattice from SDD stacking with different twist angles can change the electronic structure of 2D materials, giving them unusual transport properties like unusual superconductivity and insulating behavior. The interlayer exciton coupling can also be controlled, which makes it possible to study moiré excitons, spintronics, and valley electronics of spin-orbit coupling (Jin et al., 2019) (Seyler et al., 2019)

**FIGURE 5:**
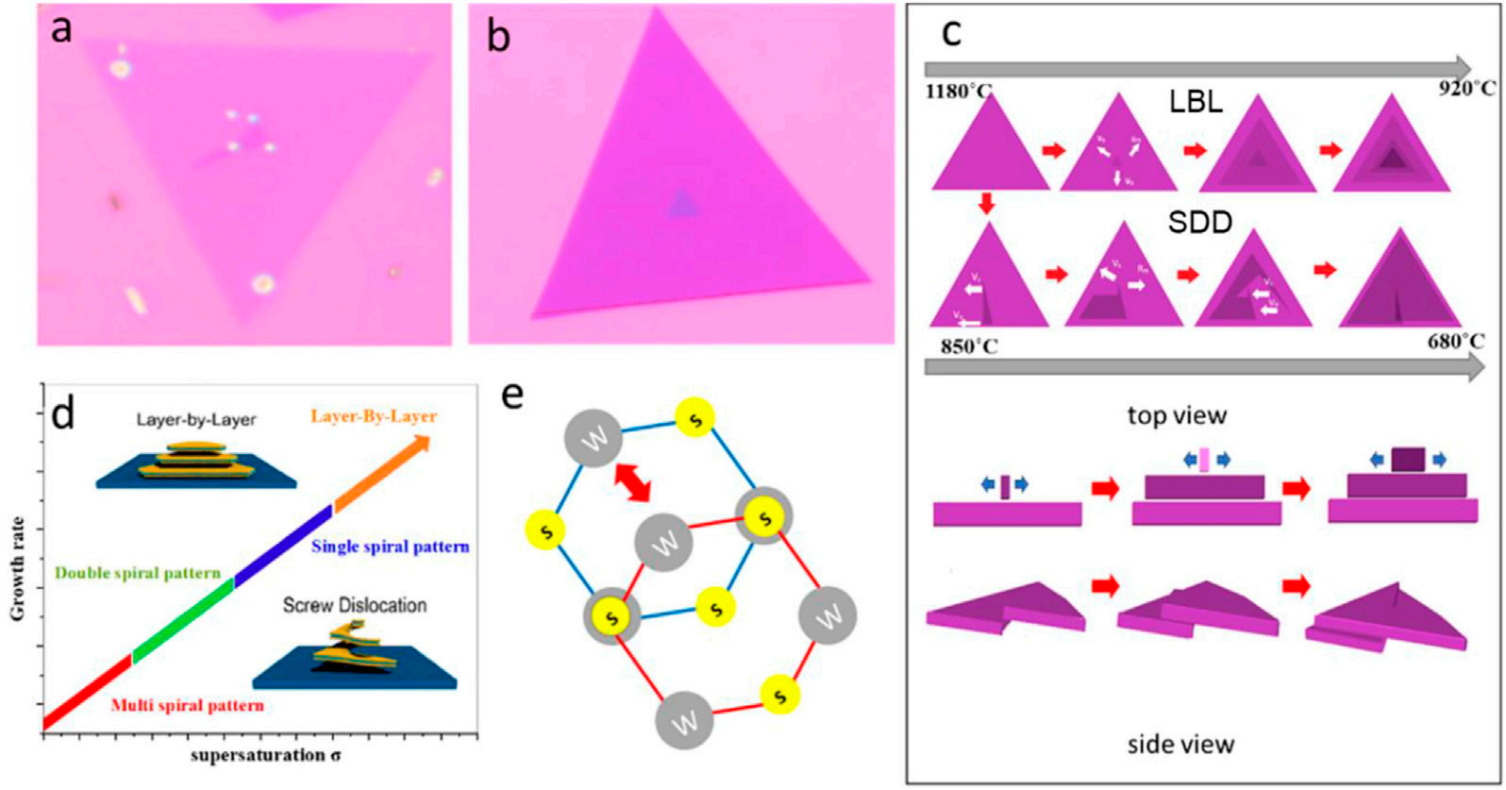
Mechanisms Formation of Layer by Layer and Dislocated WS_2_ Spirals **(A,B)** Optical image of single spiral pattern and layer by layer WS_2,_ respectively. **(C)** Schematic diagrams of Layer-By-Layer and screw dislocation propagation process. **(D)** Schematic illustrations of the growth rate of different dominant crystal growth modes as a function of supersaturation. **(E)** The tungsten atom in the top layer (red hexagon) positioned at the center of the bottom layer hexagon (blue).

The optical images in Figures 5A, B show the onset of the formation of the single helical pattern, and the LBL, respectively. It is termed anisotropic growth as it is considered the defining feature of growth almost without exception in which cells grow faster in one direction than the other, which describes the condition when the rates of growth are uneven in all directions (Figure 5A). In contrast, when growth rates are the same in all directions, growth is isotropic (LBL in Figure 5B). Figure 5C shows the model diagram of terraced structure growth. The active adsorption atoms are shown by white arrows, and the dislocation core (*v*_*c*_) depicts the axial growth rate, which is governed by the nucleation rate of new atomic layers. Outer edges (*v*_*o*_) denote the lateral growth rate, as determined by the rate at which adsorbed atoms cling to the step’s edge (Yin et al., 2015). The new surface layer progressively emerges throughout the growing phase of a terraced construction as the new atomic layer slowly nucleates on the top surface. Instead of a 1D structure growth, 2D flakes do so because the growth rate of normal to the surface (*R*_*m*_) is much slower than lateral step velocities (*v*_*s*_). When this situation occurs repeatedly, layered terraced structures will grow layer by layer. It has been discovered that the number of terraced nanosheets would directly depend on the disparity between axial and lateral development speeds. Furthermore, we translate the number of terraced into the *v*_*c*_ */v*_*o*_ ratio, which represents the ratio of axial and lateral growth rates. Furthermore, the nucleation rate of new layers on the existing top layer determines the axial growth rate. Only when the current top terraced reaches the critical size, the nucleation of the new atomic layer occurs. Therefore, the axial growth rate can be expressed as (Tersoff et al., 1994): *v*_*c*_ *= hR*_*c*_ *v*_*o*_, where h is the height of the freshly formed terraced and *R*_*c*_ is the crucial height of the top terraced at the time. According to the variations in supersaturation in the furnace, the ratios of *v*_*c*_ */v*_*o*_ and supersaturation have a linear relationship, meaning that the ratio of *v*_*c*_ */v*_*o*_ rises as supersaturation rises, which also predicts an increase in the number of terraced fields (Figure 5D). The concentration of adsorption atoms may build up, but the energy barrier at the step’s edge prevents this from happening; therefore, the nucleation of new atoms must overcome this high energy barrier (Krishnamurthy et al., 1993).Therefore, High supersaturation is crucial during the growth process of terraced structures in order to encourage the nucleation of new atomic layers and provide LBL the ability to grow at a favorable crystal growth rate. When the substrate is heated to a high temperature, spiral structures make up the majority of the nanosheets. According to Figure 1A, the nanosheets display a straightforward triangular spiral pattern, which indicates that in contrast to the low-temperature region, the screw dislocation is formed by first forming a step edge at the bottom of the growth process as the nucleation position of a new layer. Then, by continuously adsorbing the precursor atoms, results in the growth of a second layer at the top of the bottom layer, which is faster than that of the first layer.

Furthermore, the spiral structures were developed using the SDD growth mode, forming pyramid-shaped WS_2_ flakes. The Burton-Cabrera-Frank (BCF) crystal growth hypothesis provides an explanation for a number of these pyramidal growths that have been observed in 1D and 2D nanomaterials (Burton et al., 1951) (Fan et al., 2017), which states that the supersaturation of the local growth environment controls the crystal growth process. The degree of supersaturation is denoted as *σ* = In(c/c_0_), where c represents the local precursor concentration and c_0_ represents the equilibrium precursor concentration. The furnace’s temperature profile has an impact on both c and c_0_. Particularly, the deposition reaction’s thermodynamic equilibrium constant is largely governed by the local temperature, determining the value of c_0_. The precursor’s heating temperature and the precursor’s movement all dynamically affect the value of c. The deposition temperature largely affects the local supersaturation, which also affects the growth rate of nanostructures. In this theory, the system’s supersaturation drives various growth modes, including LBL and SDD growth (Woodruff, 2015). The nucleation will never be homogenous if the deposition is done on different substrates. There will always be certain defect sites that have a larger chemical potential than the rest of the substrate, which are more active for crystal development. The SDD growth mode is the most advantageous mode of development at low supersaturation, which results in spirally stacked WS_2_ pyramidal structures. These self-replicating steps of SDD layers continue the anisotropic pyramidal development in this way. Step edges (slipped planes) are produced in the bottom layer due to screw dislocations that arise under mild supersaturation conditions. Due to the large concentration of precursors, spiral development is started by the unintentional rising of a grain boundary caused by the numerous edge terming.

According to the above hypothesis, the LBL structures of WS_2_ tend to develop at the high-temperature deposition zone with a high supersaturation. In contrast, the spiral structures with screw dislocations are more likely to occur at low-temperature deposition zones with low supersaturation, where atoms may be added to the spiral step edges. Different atomic configurations are found in the top and bottom layers of the WS_2_ spiral patterns, proving the existence of a unique stacking sequence in spiral domains (Morin et al., 2011; Sarma et al., 2019). The two layers are aligned, and a lattice vector translates the upper layer. The tungsten atom in the top layer is located in the middle of the bottom layer hexagon (Figure 5E). (Zhang et al., 2014) (Ghatak et al., 2020) Additionally, toward the lower temperature region, each spiral nanostructure contains more screw dislocations than the straightforward triangular single dislocation spirals that are more commonly found in low-temperature region, suggesting that lower supersaturation condition tends to induce more complex spiral structures that correspond to multi-spiral pattern structures (Yin et al., 2015).

## Conclusion

In summary, we report the regulated growth of SDD WS_2_ nanoflakes and disclosed the underlying growth mechanisms. Through controlling the reaction temperature, different types of WS_2_ growth such as LBL and SDD growth modes can be obtained. LBL and spiral WS_2_ with ∼200 μm in size was obtained at ∼ 920°C and 850°C–680°C respectively. The interlayer coupling was weaker in the randomly twisted bilayer and at 60°C stacking than at 0°C stacking (the interlayer coupling at 60°C stacking was much weaker than the random angles.) due to the steric repulsion effect. SDD growth offers a straightforward, controllable approach to construct TMD nanostructures of various morphologies, and stackings. With a knowledge of the growth principles of stacked TMD materials, we anticipate that the comprehension of stacked TMD materials will provide attractive options for creating new materials for innovative functional nanodevices.

## Data Availability

The original contributions presented in the study are included in the article/Supplementary Material, further inquiries can be directed to the corresponding author.
