# Supplementary material for: PVD growth of spiral pyramid-shaped WS2 on SiO2/Si driven by screw dislocations
Source: Front Chem. 2023 Mar 3;11:1132567. doi: 10.3389/fchem.2023.1132567 (PMC10022673; doi:10.3389/fchem.2023.1132567)
Supplement: Supplementary file 1 [file DataSheet1.docx]

**Supporting information**

PVD Growth of Spiral Pyramid-shaped WS_2_ on SiO_2_/Si Driven by Screw Dislocations

Madoune Yassine^1, #,^ *, DingBang Yang ^1,#^, Yameen Ahmed^2^, Mansour .M. Al-Makeen^1^, , Han Huang^1^

^1^ Hunan Key Laboratory of Super-microstructure and Ultrafast Process, School of Physics and Electronics, Central South University, Changsha 410083, P. R. China.

^2^ Department of Electrical & Computer Engineering, University of Victoria, Victoria, British Columbia V8P 5C2, Canada.

* Correspondence: [madouneyassine001@csu.edu.cn](mailto:madouneyassine001@csu.edu.cn)

Synthesize WS_2_ nanoflakes includes two stages: The heating stage (including Annealing, Temperature stability, and Growth) and the cooling stage. The heating stage lasts approximately 70 minutes from room temperature to 1180 °C. The temperature in the central region of the furnace is raised from room temperature to 300 °C (annealing stage) at a rate of 15 °C / min within 20 minutes and 300 sccm of carrier gas flow at the same time. After reaching the required temperature, we adjust the temperature for 5 minutes (Temperature stability stage: the stability of the temperature inside the tube to ensure an ideal and equal distribution of the temperature inside the tube). Then the temperature was raised to 600 °C with the same steps repeated. The N_2_ flow was reduced to 60 sccm, and the temperature was raised to 1180°C at a rate of 39.3 °C/min in 20 minutes), and keep it for 12 minutes (third stage: growth stage), then PVD tube furnace cooling at under natural conditions to room temperature for ~ 40 minutes to avoid cracks in the substrate due to the sudden temperature change.
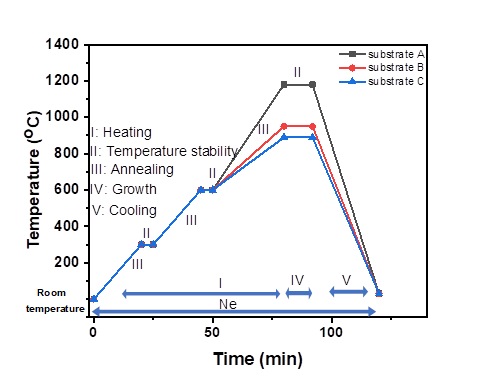


**Figure S1** Temperature setting in the process of the growth of WS_2_.


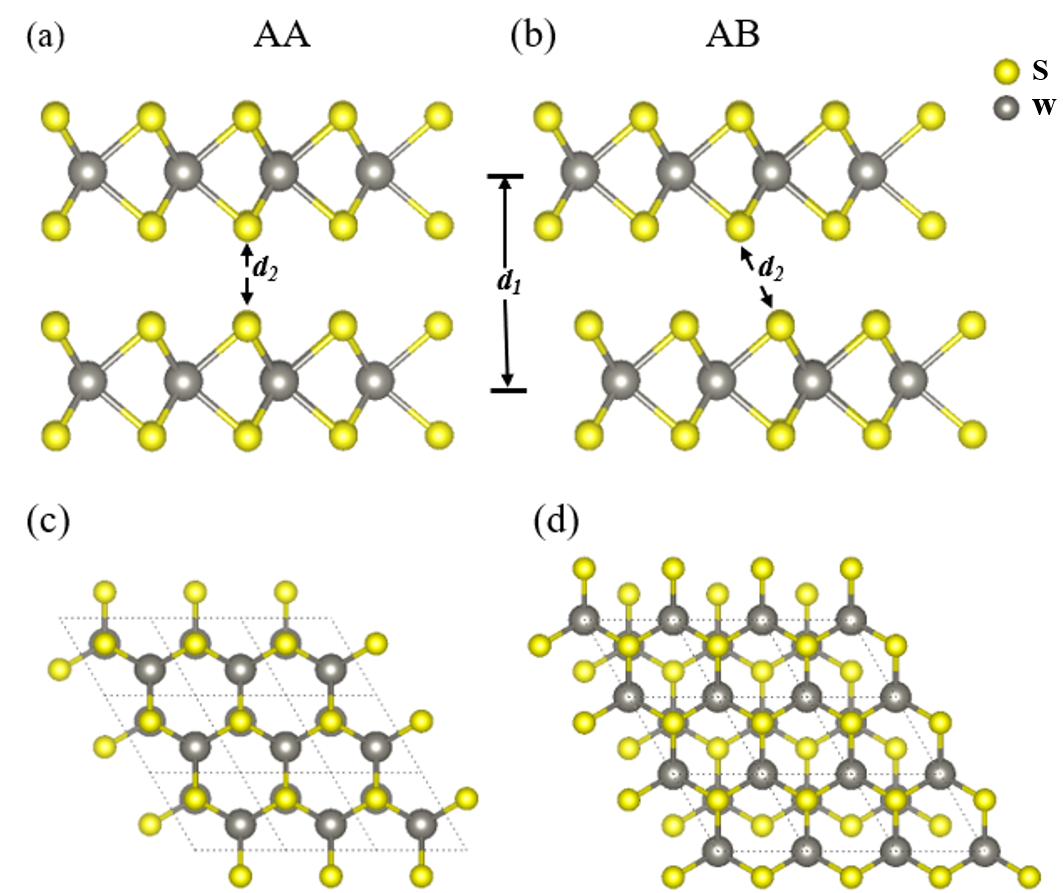


Figure S2. Atomic structure of AA stacking and AB stacking hetero-bilayer WS_2_ in a 3$\times$3$\times$1, side view (upper panel) and top view (lower panel), respectively. Large and small spheres represent the W and S atoms, respectively. Color coding is used to distinguish the different atomic species. ***d_1_*** and ***d_2_*** are the interlayer distance (W_1_-W_2_) and the bond length of (S_1_-S_2_) respectively


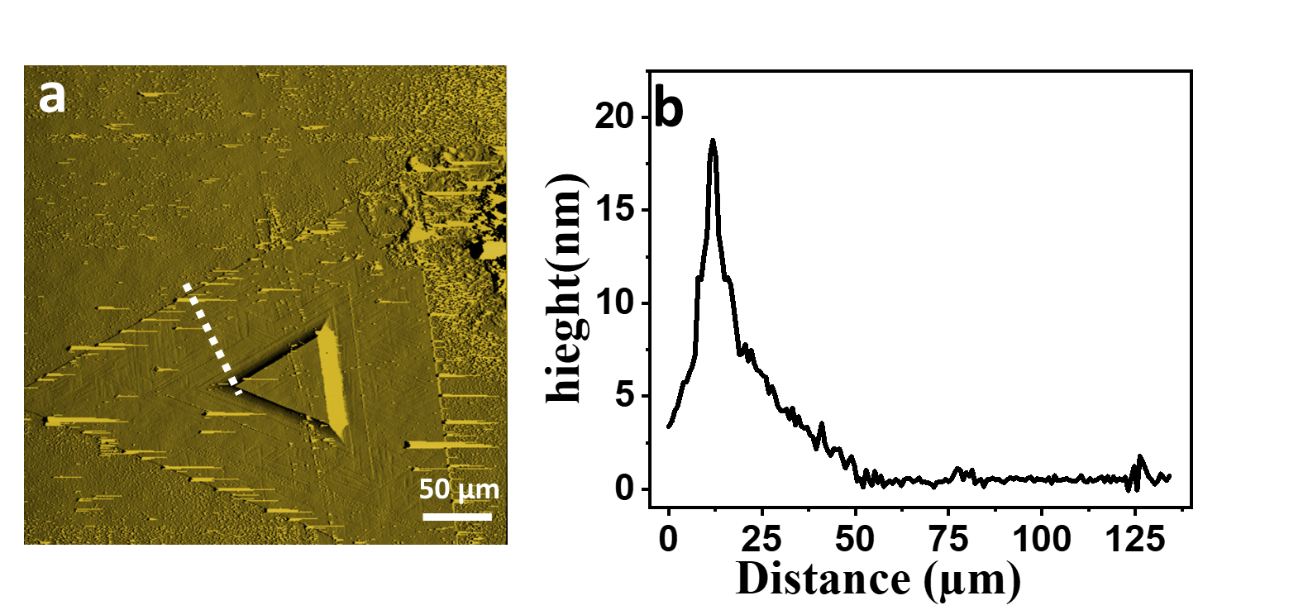


**Figure S3 | Layer by Layer (LBL) growth: AFM image of thick LBL WS_2_ flakes**. (a) AFM phase image of a thick triangular flake. (b) Profile of the white dotted line in the AFM image.

It indicates the height from edge to center is ~25 nm in the spiral WS_2_ flakes of Figure S4a.


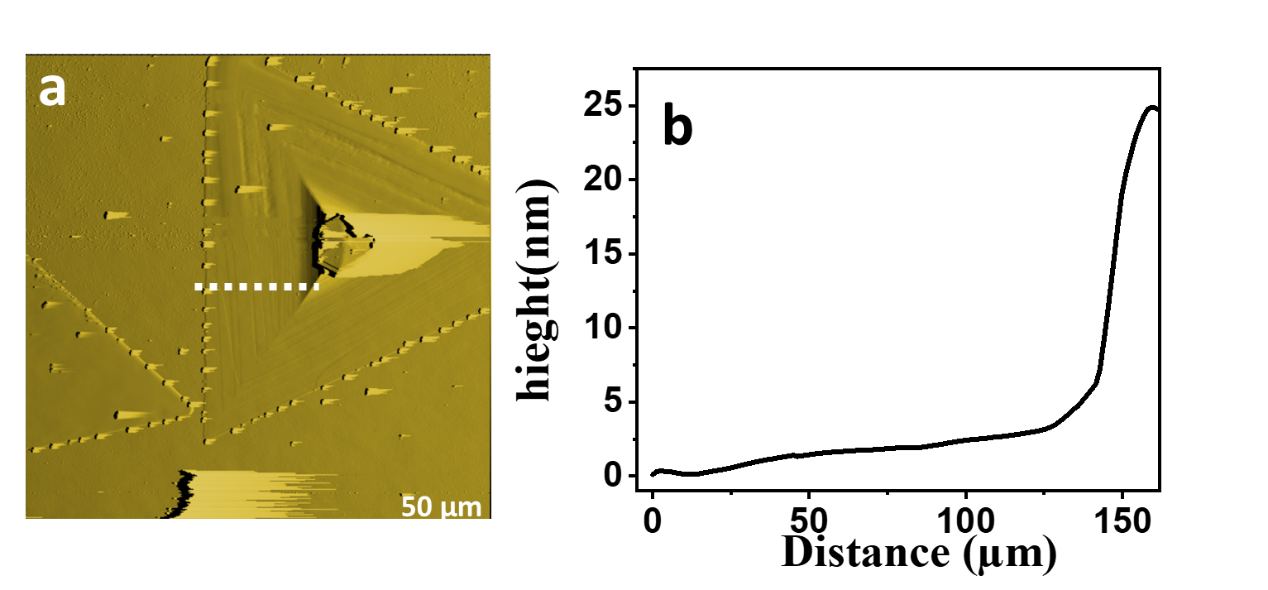


**Figure S4 | screw dislocation growth of single spiral patterns: AFM image of thick SDD WS2 flakes. (**a) AFM phase image of a thick triangular flake. (b) Profile of the white dotted line in the AFM image.

It can be confirmed that the double spiral WS_2_ with the included angles of 0˚, 120˚ and 240° and mixed spiral WS_2_ with the included angles of 60˚, 180˚, and 300˚ by AFM images. It should be noted that compared to single or double-spiral pattern structures, multi-spiral pattern nanostructures are far less often seen. (Fan et al., 2018)


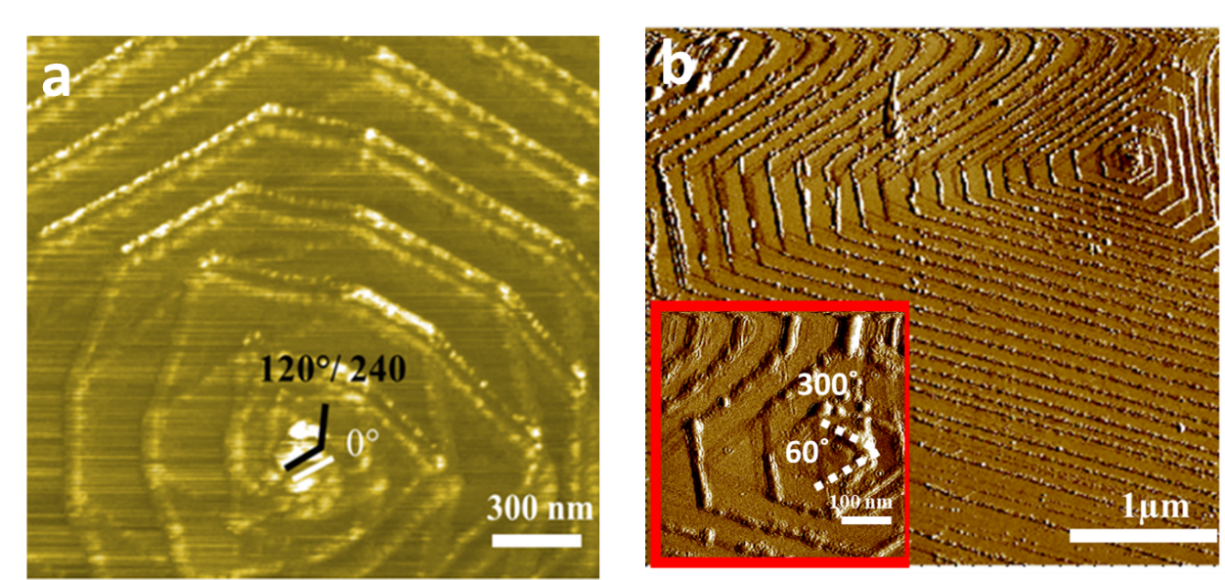


.

**Figure S5 | screw dislocation growth of different spiral patterns:** (a) AFM image of double-spiral patterns. (b) AFM image of multi-spiral patterns.


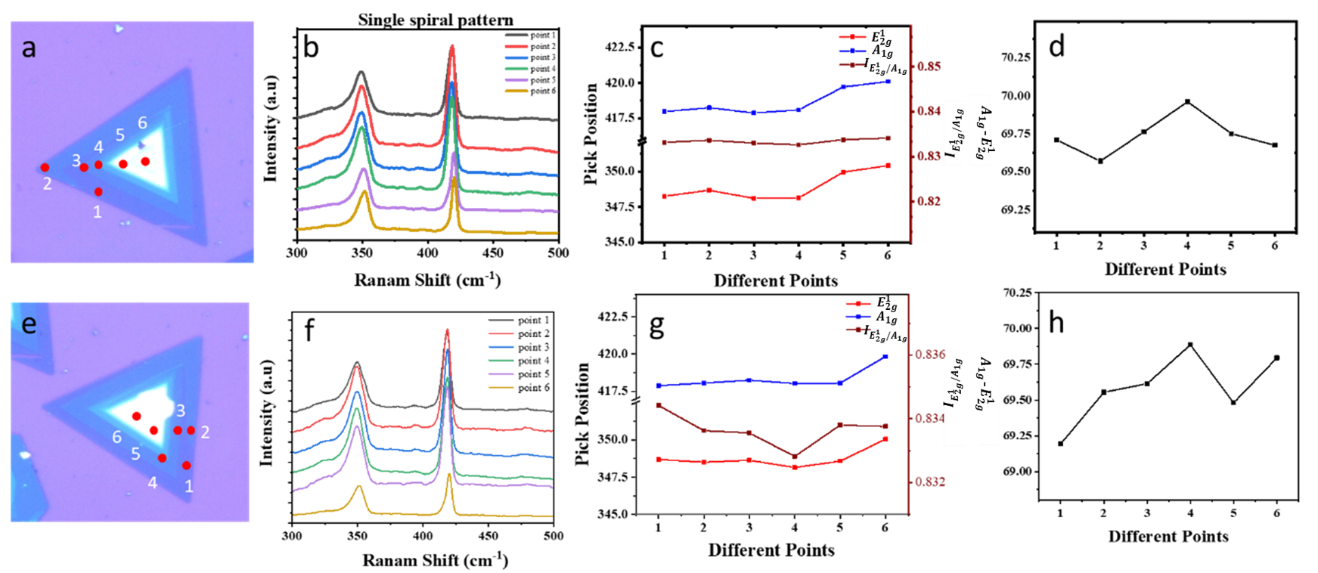


**Figure S6 | screw dislocation growth of different spiral patterns**. (a, e) Optical microscopy image of single spiral WS_2_. (b, f) Corresponding Raman spectra of the marked points in panel (a, e). (c, g) Intensity ratios, and peak frequencies were collected from regions with different ten points (marked points in panel a, e.) respectively (d, h) variation $E_{2g}^{1}$ and $A_{1g}$ on different points.


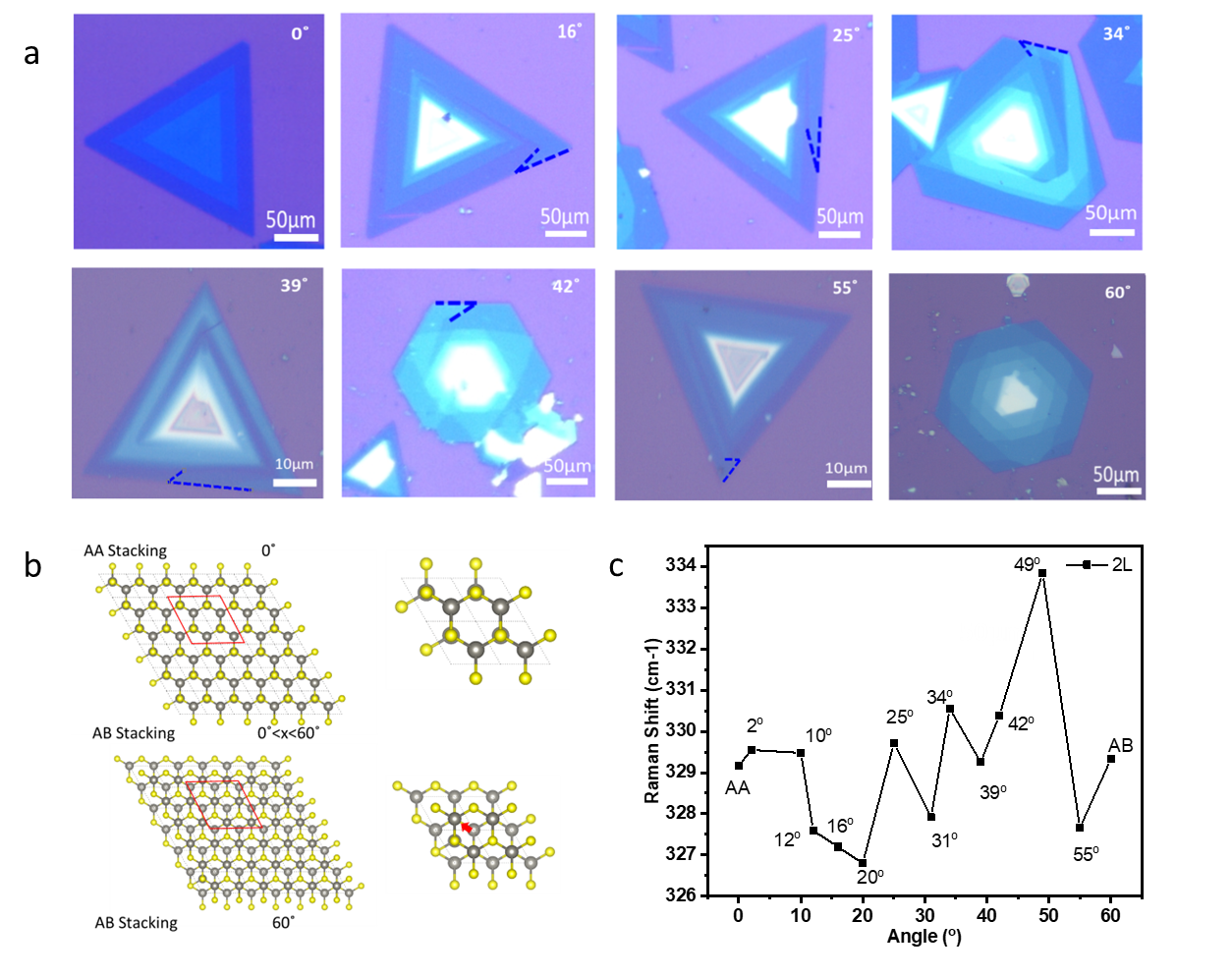


**Figure S7**. **Bilayer WS_2_ on single spiral pattern with different twist angles**. (a) Optical images and (b) General structural features of 2D-stacked MX_2_ bilayers (Top) AA stacking, (down) AB stacking. Atomic structure of hetero-bilayer stacking WS_2_ with different angles in a 2$\times$2$\times$1 supercell from a top view of AA stacking (upper panel) and top view of AB stacking (lower panel), respectively. (c) Raman Shift (2L(M) mode) at different twist angles on single spiral patterns of bilayer WS_2_. (Berkdemir et al., 2013)

**References**

Ryu, G. H., France-Lanord, A., Wen, Y., Zhou, S., Grossman, J. C., and Warner, J. H. (2018). Atomic Structure and Dynamics of Self-Limiting Sub-Nanometer Pores in Monolayer WS_2_. *ACS Nano* 12, 11638–11647. doi: 10.1021/acsnano.8b07051.

Fan, X., Zhao, Y., Zheng, W., Li, H., Wu, X., Hu, X., et al. (2018). Controllable Growth and Formation Mechanisms of Dislocated WS_2_ Spirals. *Nano Lett.* 18, 3885–3892. doi: 10.1021/acs.nanolett.8b01210.

Berkdemir, A., Gutiérrez, H. R., Botello-Méndez, A. R., Perea-López, N., Elías, A. L., Chia, C. I., et al. (2013). Identification of individual and few layers of WS_2_ using Raman Spectroscopy. *Sci. Rep.* 3, 1–8. doi: 10.1038/srep01755.
